# Analysis of clinical features and identification of risk factors in patients with non-alcoholic fatty liver disease based on FibroTouch

**DOI:** 10.1038/s41598-023-41596-2

**Published:** 2023-09-08

**Authors:** Yan Liao, Lei Liu, Jiayao Yang, Xiaoli Zhou, Xiaoli Teng, Yixi Li, Ying Wan, Jian Yang, Zhaohong Shi

**Affiliations:** 1 Department of Gastroenterology, Wuhan No.1 Hospital, Wuhan, China; 2 Department of Pancreatic Surgery, Renmin Hospital of Wuhan University, Wuhan, China

**Keywords:** Gastroenterology, Risk factors

## Abstract

Our aim was to explore the correlation between ultrasound attenuation parameter (UAP) and liver stiffness measurement (LSM) based on FibroTouch (China) and clinical features in patients with non-alcoholic fatty liver disease (NAFLD), so as to provide a certain basis for the clinical application of FibroTouch in NAFLD. Hepatic steatosis and fibrosis in patients with NAFLD were graded according to FibroTouch, and the relationship between steatosis and fibrosis levels and clinical characteristics was retrospectively analyzed. Hepatic steatosis was positively related with weight, BMI, waist, hyperlipidemia, hyperuricemia, FBG, UA, TG, ALT, AST, GGT, LSM and hepatic fibrosis grading, and was negatively related with gender (male), age and AST/ALT ratio. Hepatic fibrosis was positively related with age, BMI, waist, hypertension, FBG, ALT, AST, GGT, NFS, APRI, FIB-4, UAP and hepatic steatosis grading, and was negatively related with blood platelet (PLT) counts. Moreover, BMI, waist, TG, ALT and LSM were independent risk factors of hepatic steatosis, while decreased PLT counts, AST and UAP were independent risk factors of hepatic fibrosis. Body mass parameters, metabolic risk factors and liver function indicators increase the risk of hepatic steatosis and fibrosis in patients with NAFLD, and UAP and LSM can interact with each other.

## Introduction

Non-alcoholic fatty liver disease (NAFLD) is defined as the accumulation of triglycerides within hepatocytes that exceeds 5% of liver weight in non-alcoholics^1^, including a spectrum of diseases from simple steatosis (non-alcoholic fatty liver, NAFL) to non-alcoholic steatohepatitis (NASH) and fibrosis. The global prevalence of NAFLD is 25.24%, with 40.76% progression to fibrosis and 0.09% mean annual rate of progression in NASH^2^. The highest rates were reported from South America and the Middle East, followed by Asia, the United States and Europe^3^. The annual medical cost attributed to NAFLD exceeds €35 billion in Europe (United Kingdom, France, Germany and Italy) and $100 billion in the United States^4^.

NAFLD is the most common hepatic manifestation of metabolic syndromes and is strongly associated with obesity and insulin resistance. The change in nomenclature has been proposed from NAFLD to metabolic associated fatty liver disease (MAFLD)^5^. NAFLD has emerged as the most frequent cause of cirrhosis and hepatocellular cancer (HCC)^6^, and has been the leading cause of chronic liver disease-related mortality^3^.

Among the available non-invasive tests, transient elastography by FibroScan® (Echosens, Paris, France) is commonly used by hepatologists in Europe and Asia, and has been approved by the United States Food and Drug Administration for the diagnosis of liver disease in 2013. The controlled attenuation parameter (CAP) and liver stiffness measurement (LSM) determined by FibroScan are the evidence-based non-invasive indicators of hepatic steatosis and fibrosis^7, 8^. FibroTouch is an alternative of FibroScan developed by Wuxi Hisky Medical Technologies of China since 2010, and has been widely used in the evaluation of liver disease and liver health screening of high-risk populations in China. FibroTouch has good diagnostic performance for hepatic steatosis and fibrosis^9^.

Here, we enrolled 352 Chinese patients with NAFLD. Liver steatosis and stiffness were determined by FibroTouch and clinical characteristics were collected including demographics, body mass parameters, medical history, Laboratory parameters and noninvasive fibrosis markers. The aim was to evaluate the potential association between liver status as measured by FibroTouch and clinical features of patients with NAFLD.

## Materials and methods

### Study design

We conducted a retrospective analysis to explore the correlation between the ultrasound attenuation parameter (UAP) and LSM based on the vibration-controlled instantaneous elastography measured by FibroTouch (FibroTouch-FT5000, iLivTouch series, Wuxi Hisky Medical Technologies, China) and clinical features in patients with NAFLD, so as to provide a certain basis for the clinical application of FibroTouch in NAFLD. All methods were carried out in accordance with the Declaration of Helsinki. All experimental protocols were approved by the Medical Ethics Committee of Wuhan Integrated TCM and Western Medicine Hospital, and the ethics approval number was 2022–51. The Medical Ethics Committee of Wuhan Integrated TCM and Western Medicine Hospital waived the need for written and verbal approved informed consent from the enrolled participants because this was a retrospective observational study using only existing information.

### Participants

We enrolled 352 consecutive patients with NAFLD at Wuhan No. 1 Hospital between January 1, 2021 and August 31, 2022. Inclusion criteria included (i) being diagnosed with fatty liver disease by any of three techniques: conventional ultrasonography, CT or MRI, (ii) aged 18 to 75 years and (iii) having well-documented clinical information. The exclusion criteria included (i) presence of alcohol consumption ≥ 30 g/day for men or ≥ 20 g/day for women, (ii) presence of concomitant viral hepatitis or other liver diseases including drug-induced liver disease, hepatolenticular degeneration and autoimmune liver disease, and (iii) presence of total parenteral nutrition, inflammatory bowel disease, celiac disease, hypothyroidism, Cushing's syndrome, and other specific diseases leading to steatosis of the liver. Demographic, clinical and biochemical assessments and liver steatosis and stiffness measurements by FibroTouch were conducted within two weeks after diagnosis of NAFLD.

### Demographic, clinical and biochemical assessments

The demographic data were recorded including gender and age. Body mass parameters were collected including height, weight, body mass index (BMI), and waist. Participants who had been diagnosed with hypertension, diabetes, hyperlipidemia and hyperuricemia, or were taking relevant medication, were defined as having a history of these diseases. After an overnight fast, blood samples were collected and analyzed following standard laboratory procedures to determine the parameters including alanine transaminase (ALT), aspartate aminotransferase (AST), AST/ALT ratio, alkaline phosphatase (ALP), gamma-glutamyl transferase (GGT), serum total cholesterol (Chol), low-density lipoprotein cholesterol (LDL-C), Total serum triglycerides (TG), blood uric acid (UA), fasting blood glucose (FBG), albumin (ALB) and blood platelet counts (PLT).

### Four non-invasive fibrosis markers

Four non-invasive fibrosis markers including the NAFLD fibrosis score (NFS), aminotransferase to platelet ratio index (APRI), fibrosis-4 index (FIB-4) and BARD were computed using the available parameters^10, 11^.

### Liver steatosis and stiffness measurement by fibrotouch

FibroTouch measurements were performed by experienced and certificated physicians who had performed more than 500 examinations without knowledge of the results of ultrasonography, CT or MRI. FibroTouch was to determine liver UAP and LSM values on the basis of two-dimensional ultrasound image-guided positioning system, so that the measurement points could effectively avoid blood vessels, cysts, nodules and other factors that might interfere with the accuracy of detection. This technology measured the speed of the shear wave propagation which reflected the stiffness. Meanwhile, the energy attenuation of ultrasound signal during the propagation process was tracked, and the quantitative result of UAP was obtained which indicated the degree of liver steatosis. Each patient was placed supine with right arm raised behind his/her head and remained still during the procedure. The intercostal space was fully exposed, and three appropriate measurement points located in different intercostal spaces were selected in the liver of the same subject. The measured values with at least 10 valid measurements, a success rate of at least 60%, and an interquartile rang / median (IQR/median) of ≤ 30% were considered reliable, as suggested according to manufacturer's instructions. The median values of the 10 acceptable UAP (dB/m) and LSM (kPa) were expressed as the representative measurement of FibroTouch. According to Chinese guidelines and manufacturer's recommendations, the grading criteria for steatosis were mild steatosis (UAP ≤ 265 dB/m), moderate steatosis (265 dB/m < UAP ≤ 295 dB/m), and severe steatosis (UAP > 295 dB/m), and the grading criteria for fibrosis were F0–F1 (normal) (LSM < 6.7 kPa), F2 (mild hepatic fibrosis) (6.7 kPa ≤ LSM < 9.8 kPa), and F3 (progressive hepatic fibrosis) (LSM ≥ 9.8 kPa).

### Statistical analysis

All statistical tests were performed using IBM SPSS Statistics 25 (IBM, Armonk, NY, United States). Continuous variables were expressed as mean ± standard deviation (SD) for normal distribution, and one-way analysis of variance was used to compare differences following by Bonferroni test of pairwise comparison. Continuous variables were expressed as medians (inter-quartile range, IQR) for abnormal distribution, and Kruskar-wallis test was used to compare differences. Categorical variables were expressed as frequency (percentage). Binary variables were compared by Chi-square test and rank variables by Kruskal–wallis test following by Mann–Whitney test of pairwise comparison as appropriate. The correlation analysis was assessed using Spearman correlation analysis, and the regression analysis was assessed using ordered logistic regression. *P* value of < 0.05 was considered statistically significant.

### Ethics approval and informed consent

This is a retrospective observational study, which was reviewed and deemed exempt by medical Ethics Committee of Wuhan No.1 Hospital. This study fully protects the information and privacy of subjects. After full discussion by the Ethics Committee, informed consent can be exempted after weighing the risk benefit ratio.

## Results

### Patients characteristics

A total of 352 NAFLD patients were enrolled from January 2021 to August 2022. Baseline characteristics of the patients were shown in Table 1. The median age was 57 years (IQR 49–64), 47.2% were men, and 52.8% were women. 200 (56.8%) patients had hypertension, 70 (19.9%) had Type 2 diabetes (T_2_DM), 273 (77.6%) had hyperlipidaemia, and 138 (39.2%) had hyperuricemia based on history of diseases. Body mass parameters, laboratory parameters, and non-invasive fibrosis markers of enrolled patients were shown in Table 1.

**Table 1:** The characteristics of 352 NAFLD patients.

Characteristics (n = 352)	N (%)/mean ± SD/median (IQR)
Demographics	
Gender	
Male	166 (47.2%)
Female	186 (52.8%)
Age (years)	57 (49 ~ 64)
Body mass parameters	
Weight (kg)	69.0 (62.0 ~ 77.0)
BMI (kg/m^2^)	25.3 (23.6–27.4)
Normal BMI (18-24kg/m^2^)	100 (28.4%)
Overweight (24 < BMI < 28 kg/m^2^)	178 (50.6%)
Obesity (BMI ≥ 28 kg/m^2^)	74 (21.0%)
Waist (cm)	91 (85–96)
Medical history	
Hypertension	200 (56.8%)
Diabetes	70 (19.9%)
Hyperlipidemia	273 (77.6%)
Hyperuricemia	138 (39.2%)
**L**aboratory parameters	
PLT (× 10^9^/L)	235 ± 56
FBG (mmol/L)	5.2 (4.8–5.8)
UA (μmol/L)	366 (316–435)
Chol (mmol/L)	4.82 (4.19–5.53)
LDL-C (mmol/L)	3.23 (2.80–3.83)
TG (mmol/L)	1.74 (1.25–2.53)
ALT (IU/L)	22 (16–33)
AST (IU/L)	23 (19–28)
AST/ALT ratio	1.04 (0.81–1.28)
AST/ALT ratio ≥ 0.8	269 (76.4%)
ALP (IU/L)	88 (74–105)
GGT (IU/L)	26 (19–41)
Noninvasive fibrosis markers	
NFS	− 1.76 ± 1.28
APRI	0.25 (0.19–0.34)
FIB-4	1.23 (0.90–1.54)
BARD	2 (2–2)
FibroTouch parameters	
UAP (dB/m)	281 (264–295)
LSM (kPa)	6.2 (4.8–7.3)
Stage of hepatic steatosis	
Mild	97 (27.6%)
Moderate	168 (47.7%)
Severe	87 (24.7%)
Stage of hepatic fibrosis	
F0–F1	217 (61.6%)
F2	107 (30.4%)
F3	28 (8%)

### Assessment of hepatic steatosis and fibrosis using UAP and LSM by FibroTouch

As shown in Table 1, the mean UAP of these patients was 281 dB/m (IQR 264–295), and the mean LSM was 6.2 kPa (IQR 4.8–7.3). The distribution of the steatosis grading was as follows: mild steatosis, n = 97 (27.6%); moderate steatosis, n = 168 (47.4%); severe steatosis, n = 87 (24.7%). The distribution of the fibrosis grading was as follows: F0–F1, n = 217 (61.6%); F2, n = 107 (30.4%); F3, n = 28 (8%). The UAP and LSM values among different grading groups were significantly different (*P* < 0.001). There was a weak positive correlation between hepatic steatosis and fibrosis (r = 0.202, *P* < 0.001).

### The differences of demographics, body mass parameters, medical history, laboratory parameters, and non-invasive fibrosis markers in patients with different degrees of hepatic steatosis

As shown in Table 2, the patients in the severe steatosis group were predominantly male while the patients in the mild and moderate group were predominantly female (*P* < 0.05). Patients in the severe steatosis group were younger than those in the mild and moderate groups (*P* < 0.05). The weight, BMI and waist circumference were the highest in the severe steatosis group, followed by the moderate and mild group (*P* < 0.05). The serum TG, ALT, AST and GGT levels and LSM values of the severe steatosis group were higher than those in mild and moderate groups (*P* < 0.05). The AST/ALT ratio in the severe steatosis group were lower than those in the mild and moderate groups (*P* < 0.05). The proportion of patients with a history of hyperuricemia and the FBG levels were higher in the severe steatosis group than those in the mild group (*P* < 0.05). The serum UA levels of moderate and severe steatosis groups were higher than those of mild group (*P* < 0.05). In the severe steatosis group, F2 stage was more common, while in the mild and moderate groups, F0–F1 stage was more common (*P* < 0.05). There were no significant differences among the three groups in the proportions of patients with the history of hypertension, diabetes or hyperlipidemia, PLT counts, the levels of Chol, LDL-C and ALP in the blood (*P* > 0.05).

**Table 2:** Comparison of clinical features among NAFLD patients with different degrees of hepatic steatosis.

Characteristics	Mild steatosis (n = 97)	Moderate steatosis (n = 168)	Severe steatosis (n = 87)	*P* value
Demographics				
Gender				
Male	41 (42.3%)	69 (41.1%)	56 (64.4%)	0.001
Female	56 (57.7%)	99 (58.9%)	31 (35.6%)	
Mode	Female	Female	Male^a,b^	
Age (years)	59 (51 ~ 65)	58 (52 ~ 64)	52 (41 ~ 61)^a,b^	0.001
Body mass parameters				
Weight (kg)	64.0 (57.0 ~ 70.8)	67.3 (62.0 ~ 74.8)^a^	79.0 (73.0 ~ 95.0)^a,b^	< 0.001
BMI (kg/m^2^)	23.8 (22.5 ~ 25.2)	25.3 (23.7 ~ 26.7)^a^	29.1 (26.7 ~ 31.2)^a,b^	< 0.001
Waist (cm)	86 (82 ~ 91)	90 (85 ~ 95)^a^	98 (92 ~ 105)^a,b^	< 0.001
Medical history				
Hypertension	55 (56.7%)	95 (56.5%)	50 (57.5%)	0.99
Diabetes	14 (14.4%)	34 (20.2%)	22 (25.3%)	0.181
Hyperlipidemia	67 (69.1%)	134 (79.8%)	72 (82.8%)	0.054
Hyperuricemia	29 (29.9%)	67 (39.9%)	42 (48.3%)^a^	0.038
Laboratory parameters				
PLT (× 10^9^/L)	237 ± 56	237 ± 54	232 ± 59	0.793
FBG (mmol/L)	5.1 (4.8 ~ 5.5)	5.3 (4.9 ~ 5.9)	5.5 (4.9 ~ 6.1)^a^	0.028
UA (μmol/L)	337 (297 ~ 410)	367 (328 ~ 433)^a^	387 (326 ~ 478)^a^	0.003
Chol (mmol/L)	4.87 (4.07 ~ 5.49)	4.76 (4.20 ~ 5.49)	4.90 (4.29 ~ 5.66)	0.310
LDL-C (mmol/L)	3.22 (2.73 ~ 3.79)	3.19 (2.78 ~ 3.79)	3.31 (2.90 ~ 3.94)	0.185
TG (mmol/L)	1.55 (1.12 ~ 2.26)	1.66 (1.24 ~ 2.51)	2.07 (1.53 ~ 3.05)^a,b^	< 0.001
ALT (IU/L)	19 (15 ~ 25)	22 (16 ~ 31)	31 (19 ~ 45)^a,b^	< 0.001
AST (IU/L)	22 (19 ~ 27)	23 (19 ~ 27)	25 (19 ~ 33)^a,b^	0.004
AST/ALT ratio	1.15 (0.95 ~ 1.38)	1.05 (0.83 ~ 1.32)	0.86 (0.70 ~ 1.09)^a,b^	< 0.001
ALP (IU/L)	89 (77 ~ 108)	91 (77 ~ 109)	82 (69 ~ 100)	0.054
GGT (IU/L)	24 (17 ~ 35)	25 (19 ~ 40)	34 (22 ~ 53)^a,b^	< 0.001
FibroTouch parameters				
UAP (dB/m)	249 (237 ~ 259)	281 (274 ~ 289)^a^	306 (301 ~ 315)^a,b^	< 0.001
LSM (kPa)	6.0 (4.2 ~ 6.9)	6.0 (4.9 ~ 7.2)	6.7 (5.5 ~ 8.6)^a,b^	< 0.001
Stage of hepatic fibrosis				
F0-F1	69 (71.1%)	108 (64.3%)	40 (46.0%)	< 0.001
F2	25 (25.8%)	49 (29.2%)	33 (37.9%)	
F3	3 (3.1%)	11 (6.5%)	14 (16.1%)	
Median	F0–F1	F0–F1	F2^a,b^	

^a^*P* < 0.05 compared with mild steatosis group in pairwise comparison, ^b^*P* < 0.05 compared with moderate steatosis group in pairwise comparison.

### Correlation of hepatic steatosis with demographics, body mass parameters, medical history, laboratory parameters, and non-invasive fibrosis markers

Hepatic steatosis measured by FibroTouch showed a weak negative correlation with gender (male) and age, a moderate negative correlation with AST/ALT ratio, a weak positive correlation with hyperlipidemia, hyperuricemia, FBG, UA, TG, AST, GGT, LSM and staging of hepatic fibrosis, and a moderate positive correlation with weight, BMI, waist and ALT (*P* < 0.05) (Table 3). There was no correlation between hepatic steatosis and hypertension, diabetes, PLT, Chol, LDL-C, and ALP (Table 3).

**Table 3:** Correlation analysis between hepatic steatosis grade and various factors.

Characteristics (n = 352)	**r**	*P* value
Demographics		
Gender	− 0.154	0.004
Age(years)	− 0.171	0.001
Body mass parameters		
Weight(kg)	0.491	< 0.001
BMI(kg/m^2^)	0.583	< 0.001
Waist(cm)	0.459	< 0.001
Medical history		
Hypertension	0.005	0.92
Diabetes	0.098	0.065
Hyperlipidemia	0.121	0.023
Hyperuricemia	0.136	0.01
Laboratory parameters		
PLT(× 10^9^/L)	− 0.009	0.859
FBG(mmol/L)	0.142	0.008
UA(μmol/L)	0.176	0.001
Chol(mmol/L)	0.075	0.161
LDL-C(mmol/L)	0.079	0.137
TG(mmol/L)	0.217	< 0.001
ALT(IU/L)	0.304	< 0.001
AST(IU/L)	0.169	0.001
AST/ALT ratio	− 0.303	< 0.001
ALP(IU/L)	− 0.091	0.089
GGT(IU/L)	0.193	< 0.001
FibroTouch parameters		
LSM(kPa)	0.216	< 0.001
Stage of hepatic fibrosis	0.202	< 0.001

### Identification of risk factors for hepatic steatosis

Based on the above results, several variables including gender, age, BMI, waist, FBG, TG, ALT, AST, GGT, LSM and hyperuricemia that might affect the degree of hepatic steatosis were screened for subsequent multiple ordered logistic regression analysis. The results showed that BMI, waist, TG, ALT and LSM, but not gender, age, FBG, AST, GGT and hyperuricemia, were the independent risk factors for hepatic steatosis for NAFLD patients which significantly positively affected the degree of hepatic steatosis (*P* < 0.05) (Table 4). The higher the BMI, the greater the degree of hepatic steatosis, as well as waist, TG, ALT and LSM (*P* < 0.05) (Table 5). Among them BMI has the greatest influence on hepatic steatosis (*P* < 0.05) (Table 5).

**Table 4:** Regression analysis between hepatic steatosis grade and various factors.

Characteristics(n = 352)	B	*P* value
Age	− 0.011	0.372
BMI(kg/m^2^)	0.442	< 0.001
Waist(cm)	0.048	0.043
FBG(mmol/L)	0.085	0.232
TG(mmol/L)	0.146	0.036
ALT(IU/L)	0.039	0.003
AST(IU/L)	− 0.027	0.157
GGT(IU/L)	− 0.006	0.203
LSM(kPa)	0.123	0.025
[Gender = men]	− 0.294	0.263
[Gender = women]	0	
[Hyperuricemia = 0]	− 0.246	0.307
[Hyperuricemia = 1]	0

**Table 5:** Risk factors of hepatic steatosis screened by regression analysis.

	B	*P* value	OR	95% CI for OR	
				Lower limit	Upper limmit
BMI(kg/m^2^)	0.442	< 0.001	1.556	1.362	1.777
Waist(cm)	0.048	0.043	1.049	1.002	1.099
TG(mmol/L)	0.146	0.036	1.157	1.010	1.326
ALT(IU/L)	0.039	0.003	1.040	1.013	1.066
LSM(kPa)	0.123	0.025	1.131	1.015	1.260

### The differences of demographics, body mass parameters, medical history, laboratory parameters, and non-invasive fibrosis markers in patients with different degrees of hepatic fibrosis

The BMI, the proportions with a history of hypertension, the serum ALT and AST levels of these patients in F3 group were significantly higher than those in F0–F1 group, while the PLT counts were lower (*P* < 0.05). There was no significant difference between F0–F1 and F2 groups or between F2 and F3 groups (*P* > 0.05) (Table 6). The proportions of patients with a history of diabetes, the FBG levels and the NFS scores were higher in F3 group than those in F0–F1 and F2 group (*P* < 0.05), while there were no significant differences between F0–F1 and F2 groups (Table 6). The serum TG levels of F3 group were higher than those of F2 group (*P* < 0.05) (Table 6). There were significant differences in APRI scores, with the largest in F3 group, followed by F2 group and the smallest in F0–F1 group (*P* < 0.05) (Table 6). The GGT values, FIB-4 scores and the UAP values of F2 and F3 groups were higher than those of F0–F1 group (*P* < 0.05) (Table 6). In F0–F1 and F2 groups, the proportions of patients with moderate hepatic steatosis were the highest, while the severe hepatic steatosis was more common in F3 group (*P* < 0.05) (Table 6). There were no significant differences among the three groups in gender, age, weight, waist, the proportion of patients with the history of hyperlipidemia or hyperuricemia, the levels of UA, Chol, LDL-C, AST/ALT ratio and ALP in the blood and BARD scores (Table 6).

**Table 6:** Comparison of clinical features among NAFLD patients with different degrees of hepatic fibrosis.

Characteristics	F0-F1 (Normal) (n = 217)	F2 (Mild liver fibrosis) (n = 107)	F3 (Progressive liver fibrosis) (n = 28)	*P* value
Demographics				
Gender				
Male	99 (45.6%)	54 (50.5%)	13 (46.4%)	0.711
Female	118 (54.4%)	53 (49.5%)	15 (53.6%)	
Age (years)	57 (49 ~ 63)	59 (51 ~ 66)	60 (49 ~ 66)	0.096
Body mass parameters				
Weight (kg)	69.0 (61.0 ~ 77.3)	69.0 (62.0 ~ 77.0)	70.0 (62.0 ~ 78.0)	0.543
BMI (kg/m^2^)	25.2 (23.5 ~ 27.2)	25.5 (24.0 ~ 27.6)	26.4 (25.1 ~ 29.4)^a^	0.029
Waist (cm)	90 (84 ~ 96)	92 (86 ~ 97)	92 (88 ~ 100)	0.05
Medical history				
Hypertension	114 (52.5%)	63 (58.9%)	23 (82.1%)^a^	0.01
Diabetes	42 (19.4%)	15 (14.0%)	13 (46.4%)^a,b^	0.001
Hyperlipidemia	163 (75.1%)	85 (79.4%)	25 (89.3%)	0.205
Hyperuricemia	80 (36.9%)	46 (43.0%)	12 (42.9%)	0.523
**L**aboratory parameters				
PLT (× 10^9^/L)	241 ± 54	231 ± 54	210 ± 67^a^	0.009
FBG (mmol/L)	5.2 (4.8 ~ 5.7)	5.2 (4.8 ~ 5.8)	6.0 (5.5 ~ 7.0)^a,b^	< 0.001
UA (μmol/L)	358 (312 ~ 430)	367 (323 ~ 445)	407 (312 ~ 456)	0.403
Chol (mmol/L)	4.81 (4.19 ~ 5.51)	4.82 (4.20 ~ 5.54)	4.88 (4.14 ~ 5.77)	0.935
LDL-C (mmol/L)	3.22 (2.81 ~ 3.81)	3.24 (2.80 ~ 3.88)	3.33 (2.77 ~ 4.08)	0.919
TG (mmol/L)	1.77 (1.24 ~ 2.60)	1.56 (1.19 ~ 2.27)	2.13 (1.57 ~ 3.31)^b^	0.042
ALT (IU/L)	21 (16 ~ 29)	23 (16 ~ 38)	32 (19 ~ 57)^a^	0.004
AST (IU/L)	22 (19 ~ 27)	23 (19 ~ 31)	27 (21 ~ 46)^a^	0.002
AST/ALT ratio	1.05 (0.83 ~ 1.29)	1.00 (0.77 ~ 1.29)	1.03 (0.78 ~ 1.20)	0.582
ALP (IU/L)	89 (76 ~ 105)	88 (73 ~ 103)	86 (74 ~ 118)	0.823
GGT (IU/L)	25 (18 ~ 39)	29 (21 ~ 42)^a^	41 (24 ~ 72)^a^	0.001
Noninvasive fibrosis markers				
NFS	-1.88 ± 1.19	-1.73 ± 1.30	-1.00 ± 1.58^a,b^	0.003
APRI	0.23 (0.18 ~ 0.31)	0.27 (0.20 ~ 0.37)^a^	0.40 (0.29 ~ 0.73)^a,b^	< 0.001
FIB-4	1.14 (0.88 ~ 1.43)	1.29 (0.95 ~ 1.72)^a^	1.67 (1.10 ~ 2.51)^a^	< 0.001
BARD	2 (2 ~ 2)	2 (1 ~ 2)	2 (1 ~ 3)	0.236
FibroTouch parameters				
UAP (dB/m)	275 (259 ~ 292)	287 (267 ~ 301)^a^	297 (283 ~ 307)^a^	< 0.001
LSM (kPa)	5.2 (4.2 ~ 6.0)	7.3 (7.0 ~ 8.4)^a^	11.3 (10.4 ~ 13.3)^a,b^	< 0.001
Stage of hepatic steatosis				
Mild	69 (31.8%)	25 (23.4%)	3 (10.7%)	< 0.001
Moderate	108 (49.8%)	49 (45.8%)	11 (39.3%)	
Severe	40 (18.4%)	33 (30.8%)	14 (50.0%)	
Median	Moderate	Moderate^a^	Moderate-severe^a^	

^a^*P* < 0.05 compared with F0–F1 group in pairwise comparison, ^b^*P* < 0.05 compared with F2 group in pairwise comparison.

### Correlation of hepatic fibrosis with demographics, body mass parameters, medical history, laboratory parameters, and non-invasive fibrosis markers

Hepatic fibrosis measured by FibroTouch showed a weak negative correlation with PLT, a weak positive correlation with age, BMI, waist, hypertension, FBG, ALT, AST, GGT, NFS, APRI, FIB-4, UAP and stage of hepatic steatosis (*P* < 0.05) (Table 7). There was no correlation between hepatic fibrosis and gender, weight, diabetes, hyperlipidemia, hyperuricemia, UA, Chol, LDL-C, TG, AST/ALT ratio, ALP and BARD (Table 7).

**Table 7:** Correlation analysis between hepatic fibrosis grade and various factors.

Characteristics(n = 352)	r	*P* value
Demographics		
Gender	− 0.034	0.521
Age(years)	0.115	0.032
Body mass parameters		
Weight(kg)	0.056	0.291
BMI(kg/m^2^)	0.125	0.019
Waist(cm)	0.131	0.014
Medical history		
Hypertension	0.130	0.014
Diabetes	0.056	0.293
Hyperlipidemia	0.084	0.114
Hyperuricemia	0.059	0.266
Laboratory parameters		
PLT(× 10^9^/L)	− 0.136	0.011
FBG(mmol/L)	0.137	0.01
UA(μmol/L)	0.070	0.188
Chol(mmol/L)	0.012	0.815
LDL-C(mmol/L)	0.016	0.76
TG(mmol/L)	− 0.003	0.958
ALT(IU/L)	0.155	0.004
AST(IU/L)	0.178	0.001
AST/ALT ratio	− 0.055	0.301
ALP(IU/L)	− 0.026	0.628
GGT(IU/L)	0.195	< 0.001
Noninvasive fibrosis markers		
NFS	0.144	0.007
APRI	0.241	< 0.001
FIB-4	0.210	< 0.001
BARD	− 0.013	0.814
FibroTouch parameters		
UAP(kPa)	0.240	< 0.001
Stage of hepatic steatosis	0.202	< 0.001

### Identification of risk factors for hepatic fibrosis

Based on the above results, several variables including age, PLT count, BMI, waist, FBG, TG, ALT, AST, GGT, UAP and hypertension that might affect the degree of hepatic fibrosis were screened for subsequent multiple ordered logistic regression analysis. The results showed that PLT count, AST and UAP, but not age, BMI, waist, FBG, TG, ALT, GGT and hypertension, were the independent risk factors for hepatic fibrosis for NAFLD patients which significantly affected the degree of hepatic fibrosis (Table 8). A smaller PLT count was associated with a higher degree of liver fibrosis, while a larger AST level and UAP value were associated with a higher degree of liver fibrosis (*P* < 0.05) (Table 9). The influence of these three factors on hepatic fibrosis with NAFLD patients was statistically significant, but the effect was not strong.

**Table 8:** Regression analysis between hepatic fibrosis grade and various factors.

Characteristics (n = 352)	B	*P* value
Age	0.023	0.071
PLT(× 10^10^/L)	− 0.046	0.038
Waist	0.019	0.320
FBG(mmol/L)	0.075	0.274
TG(mmol/L)	− 0.008	0.895
ALT(IU/L)	− 0.005	0.649
AST(IU/L)	0.038	0.025
GGT(IU/L)	0.002	0.707
UAP(dB/m)	0.022	< 0.001
BMI	− 0.079	0.118
[Hypertension = 0]	− 0.269	0.271
[Hypertension = 1]	0

**Table 9:** Risk factors of hepatic fibrosis screened by regression analysis.

	B	*P* value	OR	95% CI for OR	
				Lower limit	Upper limit
PLT(× 10^10^/L)	− 0.046	0.038	0.955	0.915	0.997
AST(IU/L)	0.038	0.025	1.039	1.005	1.074
UAP(dB/m)	0.022	< 0.001	1.022	1.010	1.035

## Discussion

NAFLD is the leading cause of diffuse liver disease. Hepatic steatosis plays an important role in fibrosis progression^12^. The presence of advanced fibrosis is an important criterion for evaluating the severity of chronic liver disease. 948217 patients with NAFLD from 103 observational studies were analyzed, suggesting NAFLD significantly increased the risk of HCC (HR = 1.88[95%Cl, 1.46–2.42]), and increased HCC-related mortality risk (HR = 2.16[95% Cl, 0.85–5.5])^13^. In addition, it is well known that the degree of hepatic steatosis is linked to the metabolic syndrome and the cardiovascular risk^14, 15^. Therefore, an accurate estimation of the degree of hepatic steatosis and fibrosis is important to assess the hepatic condition of NAFLD. Histopathology is the gold standard for the diagnosis with the limitations including traumatic procedure, subjective nature and sampling variabilities^16^. The detection rate of mild hepatic steatosis (fat content > 5%) by commonly used ultrasound B-mode is low with reported sensitivity of 60.9–65%^17, 18^, and a significant intra- and inter-observer variability has been reported^19, 20^.

FibroScan has been widely used clinically to assess liver fat quantification by measuring CAP in the last decade, with liver stiffness assessment by LSM simultaneously. Validated against liver biopsy, CAP has been shown to have excellent diagnostic accuracy for detecting S1, S2, and S3 hepatic steatosis^16^. Meanwhile, it was suggested that patients with an LSM < 8 kPa was at low risk of progressive fibrosis, an LSM between 8 and 10 kPa was at intermediate risk, and an LSM ≥ 10 kPa was at high risk^21^. In patients with NASH-related cirrhosis, LSM not only is a useful tool to reflect the degree of liver fibrosis but also predicts portal hypertension, varices requiring treatment, cirrhotic complications and hepatocellular carcinoma and liver-related death^22^. As one of the transient elastography techniques, FibroTouch has a rich application experience in the diagnosis of hepatic steatosis and fibrosis in China, its effectiveness has been fully verified, and it was recommended by domestic experts consensus. With its two-dimensional image-guided positioning system, FibroTouch can avoid interference factors such as blood vessels, cysts, nodules and others, and can reduce errors caused by blind measurement, and improve detection speed, success rate and accuracy. The UAP measured by FibroTouch is calculated based on BMI variables and the spectral analysis of ultrasonic echo signals, and determines skin capsular distance which can affect the accuracy of FibroScan in patients with NAFLD. This algorithm can reduce the effect of subcutaneous fat on UAP, so FibroTouch may have certain advantages in quantifying liver fat content. The diagnosis accuracy and consistency of FibroTouch for staging fibrosis in chronic liver disease was comparable with FibroScan. There was a significant correlation (rho = 0.85, *P* < 0.001) and similar cut-off values between the FibroTouch and FibroScan for liver stiffness^23^. Compared with the internationally recognized FibroScan technology, FibroTouch still has certain limitations, and its diagnostic efficacy and threshold for different types of liver disease still need more evidence to support.

In patients with NAFLD, FibroTouch has a high diagnostic success rate, and can well distinguish the steatosis degree and fibrosis stage^9^. Validated by pathology, UAP was positively correlated with hepatic steatosis, and was significantly superior to the hepatic steatosis index, and LSM was positively correlated with degree of fibrosis and NAFLD activity score (NAS)^9^. Therefore, FibroTouch was selected to evaluate the liver condition of NAFLD patients in this study, which had a rich basis for clinical and scientific research.

The prevalence of NAFLD among adults in the general population in China is approximately 15% (6.3–27.0%)^24^. The proportion of patients with advanced fibrosis in Chinese adults with NAFLD from Hong Kong is low (3.7%)^25^. There is a lack of large epidemiological data on patients with different subtypes of NAFLD in China. Our study randomly selected patients with NAFLD diagnosed by imaging in a large general hospital in Wuhan, China. Patients with NAFL, NASH, and fibrosis were included, and patients with cirrhosis were excluded because the proportion of these patients was very small. Among these patients, 27.6% had mild steatosis, 47.4% had moderate steatosis, 24.7% had severe steatosis, 61.6% had no fibrosis, 30.4% had mild fibrosis and 28% had progressive fibrosis. The median UAP of these patients with mild, moderate and severe steatosis were 249 dB/m, 281 dB/m and 306 dB/m, respectively, and the median LSM with F0–F1, F2 and F3 were 5.2 kPa, 7.3 kPa and 11.3 kPa, respectively.

Several risk factors for NAFLD have been identified from Chinese studies, including advancing age, male gender, recent weight gain, expanding waist circumference and metabolic disorders^26^. Some studies have found that waist circumference was a more accurate predictor of fatty liver than BMI^26^. Our study found that in non-cirrhotic patients with NAFLD, risk factors for the aggravation of liver steatosis included male, younger age, weight, BMI, waist circumference, history of hyperuricemia, FBG, UA, TG, ALT, AST, low AST/ALT ratio, GGT and LSM. The risk factors for the aggravation of hepatic steatosis included BMI, waist, TG, ALT and LSM. A prospective study of 5,323 FibroScan examinations found similar results, in which factors significantly associated with CAP were BMI, metabolic syndrome and LSM, and CAP increased with the number of these parameters^27, 28^.

Previous studies and systematic reviews showed that the variables associated with NASH and progressive fibrosis in patients with NAFLD were obesity (especially visceral obesity), increasing BMI, hypertension, T2DM, AST/ALT ratio > 1 and decreased PLT count^29–31^. We found that risk factors for hepatic fibrosis in non-cirrhotic patients with NAFLD were decreased PLT count, increased AST and UAP values.

Several non-invasive fibrosis markers have been used to assess hepatic fibrosis. In NAFLD patients, the negative predictive value (NPV) of these non-invasive indicators including FIB-4 index, APRI, NFS and BARD score for advanced fibrosis was about 90%, while the positive predictive value (PPV) was not high, among which FIB-4 index had the best predictive consistency^23–34^. These non-invasive tests can reliably exclude advanced fibrosis and reduce the need for liver biopsy. In a cohort of NAFLD patients from Hong Kong and France, liver stiffness measurement by FibroScan was accurate for excluding advanced fibrosis (Kleiner stage 3–4) and performed better than non-invasive scores (AST/ALT ratio, APRI, BARD, FIB-4 and NFS)^35^. A prospective study from China showed that the diagnosis accuracy and consistency of FibroTouch for staging fibrosis in chronic liver disease was better than APRI and FIB-4^23^. Our study found that the staging of hepatic fibrosis according to FibroTouch was positively correlated with NFS, APRI and FIB-4, but the correlation was weak. The first three markers, but not BARD, differed among the different stages.

Although transient elastography techniques such as FibroTouch and FibroScan provide good assessment of hepatic steatosis and fibrosis, their accuracy is affected by several confounding factors. The factors leading to the increase of LSM include hepatic inflammation, congestion and cholestasis, alcohol and food intake^36–41^.

In conclusion, as an alternative of FibroScan, FibroTouch has good diagnostic performance for hepatic steatosis and fibrosis. Even so, there were some deficiencies in this study. Firstly, we did not expand the comparison between non-NAFLD and NAFLD. Secondly, the number of people with severe fatty liver and fibrosis included in this study was too small, which may affect the judgment of the results. Thirdly, because there were few people clinically diagnosed as liver cirrhosis caused by fatty liver in our hospital, this study does not include this kind of people, and future researchers can appropriately improve, so as to make the study more comprehensive.

## Data Availability

The datasets generated and/or analysed during the current study are available from the corresponding author on reasonable request.
